# Liver Dysfunction Explains a Substantial Proportion of Circulating cfDNA Variability in HCC: An Exploratory Study

**DOI:** 10.3390/biomedicines14071561

**Published:** 2026-07-12

**Authors:** Ioana Manea, Speranta Maria Iacob, Razvan Iacob, Alina-Veronica Ghionescu, Andrei Sorop, Roxana Elena Saizu, Daria-Ana-Arina Gheorghe, Delia Prisecariu, Simona Olimpia Dima, Liliana Simona Gheorghe

**Affiliations:** 1 Department of Gastroenterology and Hepatology, Faculty of Medicine, Carol Davila University of Medicine and Pharmacy, 020021 Bucharest, Romania; 2 Center of Excellence in Translational Medicine, Fundeni Clinical Institute, 022328 Bucharest, Romania; 3 Center for Digestive Diseases and Liver Transplant, Fundeni Clinical Institute, 022328 Bucharest, Romania

**Keywords:** hepatocellular carcinoma, circulating cell-free DNA, liquid biopsy, ALBI, PALBI, cirrhosis, biomarkers

## Abstract

**Introduction:** Circulating cell-free DNA (cfDNA) has emerged as a promising minimally invasive biomarker in hepatocellular carcinoma (HCC), with potential applications in disease detection and prognostic stratification. This exploratory study aimed to evaluate the relationship between circulating cell-free DNA (cfDNA) concentration, liver dysfunction parameters, and hepatocellular carcinoma stage in an exploratory cohort: we sought to explore the extent to which cfDNA variability may be explained by the underlying liver disease environment and whether cfDNA concentration provides incremental information beyond routinely available markers of liver reserve for the discrimination between early- and late-stage hepatocellular carcinoma. **Methods:** Sixty-four newly diagnosed HCC patients were included. Clinical, laboratory, and staging data were collected. cfDNA was isolated from plasma, confirmed by on-chip electrophoresis, and quantified by fluorimetry. Logistic regression and ROC curve analyses were performed to assess the ability of several biomarker panels to discriminate early-stage HCC (BCLC 0–A) from intermediate/advanced-stage disease (BCLC B–D). Bootstrap resampling (2000 iterations) evaluated model robustness and coefficient stability. Additional linear regression analyses explored associations between cfDNA concentration and liver dysfunction parameters. **Results:** Linear regression demonstrated that liver dysfunction parameters explained approximately 53% of cfDNA variability, while HCC stage contributed minimally after adjustment. Models incorporating albumin, bilirubin, and platelet count as individual parameters demonstrated the best discriminatory performance after adjustment for liver disease etiology, achieving AUROCs up to 0.857. The only incremental value that cfDNA concentration added to the panel was an increase to its specificity (from 79.4% to 94.1%), while reducing sensitivity by 13.4%. Bilirubin and platelet count remained the most stable predictors after bootstrap, whereas cfDNA concentration was unstable. **Limitations:** The study was limited by its small sample size, cross-sectional design, and lack of longitudinal outcome assessment. External validation in larger prospective cohorts is necessary. **Conclusion:** A substantial proportion of cfDNA concentration variability (approximately 53%) was explained by routinely available liver dysfunction parameters, whereas HCC stage had minimal contribution. These findings suggest that circulating cfDNA concentration in patients with HCC may be influenced to a greater extent by the underlying cirrhotic liver environment and hepatocyte injury than by tumor burden alone. Integrated multimarker panels combining liver reserve parameters and liver disease etiology may be of interest for minimally invasive stratification of HCC. Although cfDNA concentration increased specificity for early- versus advanced-stage disease discrimination, its incremental value was low. Further validation in larger prospective cohorts is required.

## 1. Introduction

Liver cancer has a substantial epidemiological impact worldwide, with an estimated age-standardized rate of 8.6, according to World Health Organization data [1]. Hepatocellular carcinoma (HCC) is the most prevalent malignancy of the liver and, in most cases, arises in cirrhotic patients. HCC incidence is strongly associated with old age up until 75 years of age, and the male sex is clearly predominant, even more in the European population [2]. The most common etiologies of liver disease worldwide are MASLD (59%), viral infection (38%) and alcoholic liver disease (2%) [3]. However, viral hepatitis B (HBV) seems to carry the highest risk of HCC in cirrhotic patients, although sustained efforts in vaccination against HBV have reduced the disease burden, followed by viral hepatitis C (HCV) and alcoholic liver disease (ALD) [4]. Due to the heterogeneous biological behavior of HCC (derived, in part, from the variety of etiologies of the underlying liver disease) and the frequent coexistence of impaired liver function, accurate staging is essential for therapeutic decision-making and prognostic assessment. Several staging systems have been proposed, among which the Barcelona Clinic Liver Cancer (BCLC) classification remains the most widely adopted framework in clinical practice, integrating tumor burden, liver function, and performance status to guide treatment allocation. The BCLC classification was updated in 2022 [5,6]. Moreover, in a recent study evaluating patients undergoing transarterial chemoembolization (TACE), multiple HCC staging systems were assessed for prognostic accuracy, with the Barcelona Clinic Liver Cancer classification demonstrating the best predictive performance [7].

Current therapeutic strategies for HCC include surgical resection, liver transplantation, locoregional therapies, and systemic treatment, with selection largely dependent on tumor stage and underlying hepatic reserve. In this context, reliable biomarkers and prognostic models are increasingly important for early detection and risk stratification. The GALAD score, which combines gender, age, and serum biomarkers including alpha-fetoprotein (AFP), AFP-L3, and des-gamma-carboxy prothrombin (DCP), has demonstrated promising diagnostic performance for HCC detection. Additionally, assessment of liver function plays a central role in treatment selection and prognosis. The albumin–bilirubin (ALBI) score has emerged as an objective tool for evaluating hepatic reserve and may provide improved prognostic stratification compared with traditional liver function classifications [8,9,10]. Further studies have shown that the addition of platelets may provide incremental value to the ALBI score in both stratifying the subjacent liver dysfunction and in the prognosis of HCC [11,12,13,14,15].

Circulating cell-free DNA (cfDNA) represents small fragments of double-stranded DNA, up to 200 base pairs (bp), released into the bloodstream through apoptosis, necrosis, and active secretion from both normal and tumor cells. It has emerged as a promising minimally invasive biomarker in oncology. In hepatocellular carcinoma (HCC), cfDNA-based liquid biopsy approaches have gained increasing interest due to their potential utility in early detection, prognostic stratification, treatment monitoring, and assessment of tumor dynamics. Recent studies suggest that quantitative and qualitative alterations in cfDNA may reflect tumor burden and biological behavior, supporting its potential role as an adjunctive biomarker in HCC management [16,17].

This study presents an exploratory analysis of cfDNA behavior in relation to liver dysfunction and HCC stage, incorporating routinely available clinical and biochemical parameters used in daily hospital practice. The primary objective was to evaluate the extent to which cfDNA concentration may be associated with established markers of liver reserve and with tumor staging and whether it may bring additional value in a multimarker panel for HCC stage discrimination. Given its simplicity and accessibility, cfDNA concentration was selected as the liquid-biopsy parameter of interest, while more advanced cfDNA features such as tumor-specific mutations, methylation patterns, fragmentomic profiles, and nucleosome signatures were beyond the scope of the present study.

## 2. Materials and Methods

A total of 64 patients newly diagnosed with HCC were included in the study upon referral to the Gastroenterology Unit of Fundeni Clinical Institute, Bucharest. Clinical data and biological samples included in the present analysis were obtained through the Fundeni Clinical Institute biobank between February 2021 and November 2025. The cohort comprised retrospectively collected cases (2021–2023) and prospectively enrolled cases (2024–2025). cfDNA isolation, processing, and data extraction for the present analysis were performed between 2024 and 2026. The study was approved by the Ethics Committee of Fundeni Clinical Institute (registration number 5475/02.02.2024). Patient inclusion criteria are detailed in Table 1.

The HCC diagnosis was established in the Hepatology and Liver Transplant Clinic of Fundeni Clinical Institute according to current EASL guidelines, based on contrast-enhanced computed tomography (CT) imaging findings.

Clinical data were retrieved from the institutional hospital database and included patient age, sex, diagnosis, etiology of the underlying liver disease, Child–Pugh class, BCLC stage, history of viral hepatitis infection (current or prior), viral etiology, and cirrhosis status at the time of sample collection.

Laboratory parameters extracted from the database comprised alpha-fetoprotein (AFP), carbohydrate antigen 19-9 (CA19-9), liver transaminases (ALT and AST), total bilirubin (TBIL), alkaline phosphatase (ALP), gamma-glutamyl transferase (GGT), albumin (ALB), international normalized ratio (INR), creatinine (CREA), urea, and platelet count (PLT).

For each participant, approximately 30 mL of peripheral blood was collected using EDTA (ethylenediaminetetraacetic acid) vacutainers and one clot activator vacutainer for biobanking and subsequent analyses. All samples were pre-processed within 2 h of collection.

The study protocol required that all research blood samples be collected concurrently with routine laboratory investigations requested by the attending physician. Blood collected in clot-activator tubes was centrifuged at 4000 rpm, and the resulting serum was stored at −80 °C. EDTA tubes were centrifuged at 2000 rpm to separate plasma and buffy coat fractions. The buffy coat was stored at −80 °C, while plasma samples underwent a second centrifugation step at 14,000× *g*. The supernatant was subsequently aliquoted into up to three fractions and preserved at −80 °C until circulating cell-free DNA isolation. None of the samples presented hemolysis and none of them underwent additional freeze–thaw cycles before cfDNA isolation.

Isolation of cfDNA was carried out using the QIAamp^®^ MinElute^®^ cfDNA Midi Kit (Qiagen, Hilden, Germany) according to a previously adapted protocol [18].

cfDNA quantification was performed by fluorimetry using the Qubit™ dsDNA High Sensitivity Assay Kit (Invitrogen, Thermo Fisher Scientific, Waltham, MA, USA). Fragment size distribution was evaluated by on-chip electrophoresis using the DNA 1000 Kit, High Sensitivity DNA Kit, and 2100 Bioanalyzer System (Agilent Technologies, Santa Clara, CA, USA) in order to confirm that the isolated material falls into the range of cfDNA fragment size.

Statistical analyses were conducted using Microsoft Excel (Microsoft Corp, Redmond, WA, USA) and IBM SPSS Statistics for Windows, Version 27.0 (IBM Corp., Armonk, NY, USA).

Continuous variables were tested for normality and analyzed using nonparametric methods due to non-normal distributions. Group comparisons were performed using the Mann–Whitney U test.

Logistic regression analysis was used to identify potential predictors of HCC stage and to evaluate the incremental contribution of each parameter. Receiver operating characteristic (ROC) curve analysis was applied to assess the discriminatory performance for differentiating early-stage HCC (BCLC stages 0 and A) from intermediate/advanced-stage disease (BCLC stages B to D). A combined predictive model was subsequently generated using probabilities derived from logistic regression analysis. Given the limited sample size and exploratory nature of the analysis, bootstrap resampling was performed using 2000 iterations with percentile confidence intervals in order to assess coefficient stability and model robustness. Omnibus model testing, classification performance, and goodness-of-fit statistics were additionally evaluated. Further correlations between cfDNA concentration and other parameters (including HCC stage) were performed using linear regression.

## 3. Results

### 3.1. Cohort Baseline Characteristics

A total of 64 patients with hepatocellular carcinoma were included in the study cohort. The mean age of the study population was 60.5 ± 9.9 years, with ages ranging from 28 to 78 years. There was a clear predominance of the male sex (82.8%) within the cohort, slightly higher than the reported World Health Organization data for Romania [1].

### 3.2. Etiology of Underlying Liver Disease

Viral-related liver disease represented the predominant etiology, accounting for 54.7% (*n* = 35) of cases, followed by alcohol-related liver disease in 20.3% (*n* = 13) and combined alcohol- and viral-associated liver disease in 14.1% (*n* = 9). Other etiologies such as cholestatic, MASLD and cryptogenic were grouped in one category and represented 10.9% (*n* = 7) of all cases. Overall, viral hepatitis infection was present in 68.8% (*n* = 44) of patients. Regarding viral etiology, hepatitis C virus infection was identified in 28.1% (*n* = 18) of cases and hepatitis B virus infection in 21.9% (*n* = 14), while HBV + HDV coinfection was present in 18.8% (*n* = 12) of patients.

### 3.3. Severity of Liver Disease

Nearly all patients included in the cohort had cirrhosis (98.4%, *n* = 63). According to the Child–Pugh classification, most patients demonstrated preserved hepatic functional reserve, with 62.5% (*n* = 40) classified as Child–Pugh A, whereas 26.6% (*n* = 17) and 10.9% (*n* = 7) were categorized as Child–Pugh B and C, respectively.

### 3.4. Tumor Staging Characteristics

Tumor staging was evaluated according to the Barcelona Clinic Liver Cancer (BCLC) classification. Early-stage disease predominated, with 42.2% (*n* = 27) of patients classified as BCLC stage A and 10.9% (*n* = 7) as stage 0. Intermediate-stage disease (BCLC B) was observed in 21.9% (*n* = 14) of patients, while 17.2% (*n* = 11) and 7.8% (*n* = 5) were classified as BCLC stages C and D, respectively. Vascular invasion was identified in 18.8% (*n* = 12) of cases, whereas extrahepatic metastases were present in 11.1% (*n* = 7) of patients. Overall, 53.1% of patients were classified as having early-stage disease, whereas 46.9% presented with intermediate or advanced-stage HCC.

### 3.5. Initial Assessment of cfDNA Concentration in Early- Versus Late-Stage HCC Discrimination

Due to non-normal distributions, continuous laboratory variables are reported as medians and interquartile ranges (IQR). The detailed panel can be consulted in Table 2.

Circulating cfDNA fluorimetric analysis revealed a median concentration of 17.36 ng/mL plasma (IQR: 11.06–28.90). The median fragment size was 169 bp (IQR: 166–171).

Distribution analysis demonstrated substantial skewness for both parameters, supporting the use of nonparametric statistical methods.

A panel of several markers was compiled in order to account for the following: cholestasis (TBIL), liver synthetic reserve (ALB), portal hypertension (PLT) and tumor burden (cfDNA concentration). They were tested both as individual parameters inside the panel and as previously established scores (ALBI and PALBI). The composition of each panel can be consulted in Table 3.

Given the most predominant etiologies in our cohort (viral and ALD) and the differences in the pathophysiology of liver disease between them, we decided to adjust the statistical analysis by etiology of liver disease [19,20,21,22]. Logistic regression was performed on all models with and without adjustment for etiology in order to assess their relation to HCC progression. Early stage was defined as BCLC 0 or A, and late stage was defined as BCLC B-D. The results are presented in Table 4.

Bootstrap resampling (2000 iterations) was performed for all logistic regression models, with all omnibus tests of model coefficients remaining significant (*p* < 0.001). Across baseline and etiology-adjusted models, combinations incorporating liver reserve parameters consistently demonstrated the strongest discriminatory performance (models 3 and 5 achieved the best overall performance). Their performance was significantly improved after adjustment for etiology. Across those two models, platelets and bilirubin remained strong independent predictors, retaining their stability after bootstrap: in model 3, bilirubin retained a *p* = 0.007, CI [0.707; 4.676] and platelets retained a *p* = 0.004, CI [0.005; 0.042]; in model 5, bilirubin had a *p* = 0.005, CI [0.814; 4.619] and platelets had a *p* = 0.004, CI [0.007; 0.042]. Albumin survives bootstrap for model 5 with a *p* = 0.025 and CI [−3.33; −0.2], but not for model 3, where it became highly unstable: *p* = 0.088, CI [−3.32; 0.40]. cfDNA concentration also lost stability after bootstrap, with a *p* = 0.245 and CI [−0.032; 0.128], but to a lesser degree than albumin.

### 3.6. Liver Dysfunction Parameters Explain a Substantial Proportion of cfDNA Concentration Variability

Because the cfDNA-containing model did not improve overall AUROC compared with the corresponding liver-function-only model but altered the balance between sensitivity and specificity, we further explored how circulating cfDNA concentration was itself associated with routinely available markers of liver dysfunction. This analysis was intended to clarify whether total cfDNA correlated with BCLC stage independently or whether it was substantially influenced by the underlying liver disease environment. We decided to perform multiparametric linear regression with cfDNA as the dependent and the following covariates: albumin, bilirubin, platelet count and BCLC stage expressed in a binary manner (0 = early-stage; 1 = later stages). We retained the same adjustment as in the logistic regression models (etiology of the underlying liver disease), creating a new variable for each etiology: ALD, viral, ALD + viral and other. For consistency of the analysis, we used the same reference category as in the logistic regression models (ALD). Bootstrap was again performed using 2000 iterations. The overall model revealed a *p* < 0.001; R^2^ = 0.53; adjusted R^2^ = 0.47. The only component that maintained a significant *p* after bootstrap was the platelet count (*p* = 0.049), but the CI crossed 0 (−0.12; 0.257). BCLC stage collapsed after bootstrap: *p* = 0.556, CI [−9.27; 16.93]. After removing the BCLC stage from the panel and performing the same statistical analysis, the explanatory performance of the panel barely changed: *p* < 0.001; R^2^ = 0.527; adjusted R^2^ = 0.477. All bootstrap resampling coefficients, as well as linear regression performance, are detailed in Supplementary Tables S1–S3. Additionally, to assess whether any collinearity was observed between the variables included in the linear regression, the variance inflation factors (VIFs) and tolerances were calculated. No relevant multicollinearity was observed; the results are presented in Table 5.

As an additional assessment of sample adequacy, post hoc observed power estimates were calculated. The linear regression model demonstrated an observed power of 1.000, whereas the multivariable staging models 3 and 5 demonstrated observed powers of 0.920 and 0.935, respectively. Given the exploratory nature of the study, bootstrap resampling remained the primary method used to evaluate model stability and robustness, while post hoc power estimates were interpreted as complementary measures of the observed effect sizes.

## 4. Discussion

This exploratory study investigated the potential relationship between cfDNA concentration, liver dysfunction parameters and hepatocellular carcinoma stage using multivariable linear and logistic regression analyses. The primary objective was to determine whether total cfDNA concentration is more strongly associated with HCC stage or with the underlying liver disease environment and thus whether it might present potential added value in discriminating between early- and late-stage HCC. An important consideration in marker selection was clinical feasibility: routinely available laboratory parameters were chosen because they are widely accessible, while cfDNA concentration was selected as a liquid-biopsy parameter that is inexpensive and readily obtainable during standard cfDNA workflows.

The BCLC staging system was dichotomized according to the clinically relevant boundary between early-stage disease, where curative-intent strategies may be considered, and intermediate/advanced disease. Individual tumor burden variables were intentionally not reintroduced as predictors in the primary multivariable models for two reasons: 1. In order to avoid structural circularity, the BCLC staging system intrinsically incorporates anatomical tumor burden, hepatic functional reserve, and performance status. 2. Only 12 cases had vascular invasion, and 7 had extrahepatic metastases, of which 6 cases had both.

In their review on hepatocellular carcinoma, Llovet et al. present in greater detail the heterogeneous etiologies of cirrhosis and the different molecular mechanisms that promote hepatocarcinogenesis [23]. The mechanisms behind alcohol-related liver injury and viral infection-related liver injury are well documented in the literature: while alcoholic liver disease is driven mainly by direct ethanol toxicity, lipid peroxidation and oxidative stress, HBV-induced alterations disrupt apoptosis and promote oncogenesis through several molecular pathways, including but not limited to the promotion of epithelial-to-mesenchymal transition and non-coding RNA expression [24,25,26,27]. Therefore, etiology was included as an adjustment covariate because different liver disease etiologies may influence both tumor biology and biomarker expression. In our cohort, all models performed better after etiology adjustment, suggesting that underlying liver disease etiology might be a confounder.

Two multimarker panels emerged as potentially of analytical interest for future studies: model 3 (albumin, bilirubin, platelet count and cfDNA concentration) and model 5 (albumin, bilirubin and platelet count). Despite the overall model performance being nearly identical (AUROCs 0.854 and 0.857, respectively; overlapping CIs), there is a marked difference in their sensitivity–specificity balance. The model, including cell-free DNA concentration, had reduced sensitivity but revealed a 94.1% specificity in discriminating early versus late-stage HCC. Notably, the cohort was predominantly composed of patients with preserved hepatic function (Child–Pugh A: 40/64) and early-stage disease (>50% BCLC 0–A), suggesting that the observed discriminatory performance was not solely driven by severely decompensated or extensively advanced cases. Interestingly, in our cohort, the panels using the established PALBI score showed lower performance than the panels using the separate biomarkers (albumin, bilirubin and platelet count). This finding may reflect the small sample size of our cohort or simply emphasize that different cohorts may not align perfectly with the coefficients derived from the original PALBI study.

However, the interpretation of total cfDNA concentration in HCC remains complex. As summarized in reviews of liquid biopsy applications across multiple solid tumors in general and in HCC in particular, most translational and clinical interest has focused on circulating tumor DNA rather than total cfDNA concentration. This distinction is important, because total cfDNA represents a composite signal derived from both malignant and non-malignant sources. These reviews also emphasize that quantitative cfDNA assessment alone has limited biological specificity and may be strengthened by integration with additional circulating, epigenetic, fragmentomic, or clinical markers [28,29]. In line with this concept, an HBV-related HCC study including 48 patients reported that total plasma cfDNA concentration was associated not only with tumor-related features, such as tumor diameter, vascular invasion, TNM stage, and BCLC stage, but also with liver disease-related parameters, including Child–Pugh class, viral load, cirrhosis status, albumin, and prothrombin time. In multivariable analysis, tumor diameter, vascular invasion, and Child–Pugh class remained associated with elevated total cfDNA concentration, suggesting that both tumor burden and hepatic functional impairment may contribute to the circulating cfDNA pool. Our findings extend this investigation in a cohort with predominantly cirrhotic HCC patients (63 cirrhotic/64 cases). In order to estimate the extent to which cfDNA concentration may be a measure of overall liver function, we used cfDNA concentration as the dependent variable and evaluated its correlation to albumin, bilirubin, and platelet count with and without HCC stage. For consistency, the binary coding of HCC stage and etiology adjustment were identical to previous analyses. Despite the small sample size, overall model fit remained similar after bootstrap resampling, although individual coefficient stability was limited. The results suggested that the explanatory performance of the liver-function panel was relatively stable, whereas individual predictors showed limited coefficient-level robustness. Adding the BCLC stage did not improve the explanatory capacity of the liver-function model for cfDNA concentration. In both assessments, the explanatory performance of liver dysfunction remained approximately 53%. Moreover, because BCLC itself incorporates liver function, disentangling tumor burden from hepatic reserve is intrinsically difficult; however, the low contribution of HCC staging in the linear regression supports the interpretation that the associations observed for models 3 and 5 are not merely overlaps with the liver function component of the BCLC classification. Furthermore, VIF and tolerance analysis confirmed that collinearity within the linear regression was negligible.

Several reviews reveal the inconsistent performance of different cfDNA parameters (including cfDNA concentration and cfDNA mutation analyses) across previous studies [28,29,30]. Moreover, a more recent review on liquid biopsy in HCC patients underlines the paucity of circulating tumor DNA (ctDNA) in total cfDNA (<1%, even <0.01% in early stages of cancer) and that current methods of ctDNA enrichment underperform [31]. The most notable finding of the present study is that approximately 53% of the variability in cfDNA concentration could be explained by routinely available liver dysfunction parameters. In contrast, the HCC stage contributed minimally after adjustment and showed statistical instability following bootstrap validation. This observation suggests that circulating cfDNA concentration in patients with HCC may be substantially influenced by the underlying cirrhotic liver environment and hepatocyte injury rather than by tumor burden alone.

Interestingly, a recent study investigating the role of renal and liver function on the concentration of cfDNA in 846 stage I–III colorectal cancer patients observed that increased bilirubin levels were correlated with increased total cfDNA concentration levels but did not seem to influence ctDNA concentration. Bilirubin was the only tested parameter with a statistically significant correlation [32]. This observation raises two major questions: 1. Does liver dysfunction contribute to additional release, impaired clearance of cfDNA, or both? 2. Does the increase in total cfDNA concentration, but not in ctDNA concentration, further dilute the tumoral DNA in the sample? In contrast, our study focused on liver reserve parameters covering multiple aspects of liver pathophysiology (synthesis, cholestasis and portal hypertension) in a deeply altered liver environment (cirrhosis), while revealing an even stronger connection between impaired liver function and cfDNA concentration, albeit in a smaller cohort. Such an interpretation may help explain the heterogeneous performance of cfDNA parameters reported across previous HCC studies, where varying degrees of liver dysfunction may act as an important biological confounder. Together, these data support the view that total cfDNA concentration should not be interpreted as a direct surrogate of tumor burden in HCC but rather as a biologically mixed signal influenced by both tumor-related and non-tumoral liver injury-related processes and that total cfDNA concentration alone may have limited value as a biomarker of HCC stage.

## 5. Limitations

Several limitations of the present study should be acknowledged. Firstly, although the observed performance of the proposed panels was encouraging, the limited sample size may increase susceptibility to statistical instability in multivariable modeling and introduce a certain degree of optimism bias. Consequently, the identified associations should be interpreted cautiously and considered exploratory rather than definitive. Future external validation in an independent, multicentric cohort is warranted. Secondly, the cross-sectional design of the study did not permit longitudinal evaluation of clinically relevant outcomes such as disease progression, treatment response, disease-free survival, or overall survival. As a result, the prognostic significance and temporal stability of the proposed panels remain to be determined through prospective follow-up analyses. Moreover, the addition of cfDNA concentration may prove redundant in a future study focusing on sensitivity, rather than specificity. Furthermore, although adjustment for etiology was explored, the complex biological interplay between tumor burden, cirrhosis severity, systemic inflammation, and hepatocyte turnover cannot be fully disentangled within the constraints of the present design. Beyond this, while the predominance of Child–Pugh A patients and early-stage HCC reduce the likelihood that the observed signal was driven exclusively by advanced-stage hepatic dysfunction, larger prospective studies will be necessary to clarify the relationship between cell-free DNA release and the complex pathophysiology of the cirrhosis–hepatocellular carcinoma environment.

## 6. Conclusions

The present exploratory analysis suggests that liver dysfunction parameters remain the principal drivers of stage discrimination in this cohort. Importantly, approximately half of the observed cfDNA variability was explained by markers of liver dysfunction, supporting the hypothesis that the cirrhotic liver environment may be a major determinant of circulating cfDNA concentration in patients with HCC. These observations may bring additional information to the complex issue of circulating cell-free DNA variation within the context of altered liver functional reserve in relation to hepatocellular carcinoma.

The observed discriminatory performance of multimarker panels accounting for cholestasis, liver synthetic reserve and portal hypertension suggests potential utility in stratifying patients with hepatocellular carcinoma, particularly in settings where rapid or minimally invasive additional stratification may be clinically advantageous. In this cohort, the only incremental contribution of cfDNA concentration was an increase in specificity. Equally important, cfDNA concentration measurement is an integral component of any advanced cfDNA workflow, while liver reserve parameters are routinely tested in the clinical laboratory and are easily available for integration into the research dataset. As such, the association between liver injury and the concentration of cfDNA, ideally, with simultaneous ctDNA measurements may warrant further exploration in larger, multicentric cohorts.

## Figures and Tables

**Table 1:** Patient inclusion criteria.

Newly diagnosed HCC
No invasive maneuver performed on the liver before blood collection
Underlying liver disease
No history of other malignancies

**Table 2:** Paraclinical parameters of the cohort.

Laboratory Parameter	Median (IQR)
Albumin (g/dL)	4.02 (3.51–4.59)
ALP (U/L)	125.0 (87.06–162.0)
ALT (U/L)	43.0 (26.5–64.0)
AST (U/L)	46.0 (37.3–77.5)
Creatinine (mg/dL)	0.90 (0.76–1.10)
GGT (U/L)	56.0 (26.0–107.5)
INR	1.23 (1.12–1.41)
Platelets (×10^3^/µL)	128.0 (85.0–179.5)
Total bilirubin (mg/dL)	1.30 (1.00–1.98)
Urea (mg/dL)	35.45 (28.53–45.90)

**Table 3:** Panels included in the statistical analysis.

Panel	Parameters
Model 1	ALB + TBIL + cfDNA concentration
Model 2	ALBI + cfDNA concentration
Model 3	ALB + TBIL + PLT + cfDNA concentration
Model 4	PALBI + cfDNA concentration
Model 5	ALB + TBIL + PLT
Model 6	PALBI

**Table 4:** Performance of each model as predictor of early- versus late-stage HCC.

Adjustment	Model	AUROC (95% CI)	Youden Index	Sensitivity	Specificity
Baseline	Model 1	0.740 (0.615–0.865)	0.453	60%	85.3%
	Model 2	0.749 (0.627–0.871)	0.461	66.7%	79.4%
	Model 3	0.806 (0.696–0.917)	0.571	60%	97.1%
	Model 4	0.775 (0.656–0.893)	0.553	70%	85.3%
	Model 5	0.810 (0.701–0.919)	0.549	66.7%	88.2%
	Model 6	0.777 (0.661–0.894)	0.520	66.7%	85.3%
Etiology-adjusted	Model 1	0.796 (0.688–0.904)	0.475	53.3%	94.1%
	Model 2	0.785 (0.671–0.900)	0.516	63.3%	88.2%
	**Model 3**	**0.854** **(0.763–0.945)**	**0.575**	**63.3%**	**94.1%**
	Model 4	0.809 (0.702–0.916)	0.578	66.7%	91.2%
	**Model 5**	**0.857** **(0.767–0.946)**	**0.561**	**76.7%**	**79.4%**
	Model 6	0.800 (0.692–0.908)	0.504	53.3%	97.1%

**Table 5:** Multicollinearity assessment for the linear regression.

Parameter	Tolerance	VIF
Albumin	0.608	1.645
BCLC	0.723	1.383
Etiology ALD + VIR	0.659	1.517
Etiology Other	0.665	1.504
Etiology VIR	0.536	1.867
Platelet count	0.846	1.181
Total bilirubin	0.688	1.453
Dependent variable = cfDNA concentration		

## Data Availability

The original contributions presented in this study are included in the article/Supplementary Material. Further inquiries can be directed to the corresponding author.

## Supplementary Materials

The following supporting information can be downloaded at https://www.mdpi.com/article/10.3390/biomedicines14071561/s1, Table S1: Etiology-adjusted linear regression models for cfDNA concentration; Table S2: Etiology-adjusted linear regression models for cfDNA concentration; Table S3: Logistic regression models’ overall performance and component stability.
