# Supplementary material for: Liver Dysfunction Explains a Substantial Proportion of Circulating cfDNA Variability in HCC: An Exploratory Study
Source: Biomedicines. 2026 Jul 12;14(7):1561. doi: 10.3390/biomedicines14071561 (PMC13407311; doi:10.3390/biomedicines14071561)
Supplement: Supplementary file 1 [file biomedicines-14-01561-s001.zip › biomedicines-4353017-supplementary.pdf]

Supplementary Table S1 - Etiology-adjusted linear regression models for cfDNA concentration								
	Model including BCLC stage				Model excluding BCLC stage			
**Variable**	**B (SE)**	**p-value**	**95% CI**	**Bootstrap stability**	**B (SE)**	**p-value**	**95% CI**	**Bootstrap stability**
**Constant**	61.105 (21.656)	0.011	10.14 to 96.07	Stable	65.178 (20.27)	0.004	17.2 to 97.67	Stable
**Etiology: ALD + viral**	−17.771 (13.280)	0.194	−41.3 to 11.07	Unstable	−17.486 (13.318)	0.194	−41.13 to 11.34	Unstable
**Etiology: Other**	−31.443 (12.941)	0.44	−58.65 to −8.4	Stable	−30.353 (12.784)	0.045	−57.72 to −7.54	Stable
**Etiology: Viral**	−23.679 (9.038)	0.037	−40.7 to -5.99	Stable	−22.840 (8.946)	0.038	−39.54 to −5.21	Stable
**BCLC stage (0/A vs B–D)**	4.362 (6.700)	0.556	−9.27 to 16.93	Unstable	—	—	—	—
**Platelet count**	0.168 (0.083)	0.049	-0.012 to 0.257	Borderline stability	0.175 (0.077)	0.032	0.005 to 0.26	Stable
**Total bilirubin**	2.125 (3.068)	0.264	−0.03 to 11.231	Borderline stability	2.312 (3.031)	0.244	−0.24 to 11.32	Borderline stability
**Albumin**	−11.561 (5.704)	0.076	−20.3 to −2.1	Stable	−12.591 (5.043)	0.027	−20.54 to −0.62	Stable

Reference etiology category: alcohol-related liver disease (ALD).
BCLC stage coded as early-stage (0/A) versus intermediate/advanced-stage disease (B–D).
Bootstrap confidence intervals generated using 2000 resamples.

Supplementary Table S2 - Etiology-adjusted linear regression models for cfDNA concentration		
**Parameter**	**With BCLC stage**	**No BCLC stage**
**R**	0.728	0.726
**R^2**	0.530	0.527
**Adjusted R^2**	0.472	0.477
**Standard error of estimate**	23.09	22.97
**F-statistic**	9.033	10.580
**Overall model p-value**	<0.001	<0.001

Supplementary Table S3 – Logistic regression models overall performance and component stability

Model	Omnibus Model p	Nagelkerke R^2	Overall Accuracy	Components	Bootstrap p (CI)	Interpretation
**Etiology-Adjusted Model 3**	<0.001	0.537	75.0%	**Albumin**	0.088 (-3.327; 0.402)	Unstable
				**Bilirubin**	0.007 (0.707; 4.676)	Stable
				**Platelet count**	0.004 (0.005; 0.042)	Very stable
				**cfDNA concentration**	0.245 (-0.032; 0.128)	Moderate instability
**Etiology-Adjusted Model 5**	<0.001	0.520	76.6%	**Albumin**	0.025 (-3.331; -0.17)	Borderline stable
				**Bilirubin**	0.005 (0.814; 4.619)	Stable
				**Platelet count**	0.004 (0.007; 0.042)	Very stable

Reference etiology category: alcohol-related liver disease (ALD).
BCLC stage coded as early-stage (0/A) versus intermediate/advanced-stage disease (B–D).
Bootstrap confidence intervals generated using 2000 resamples.
